# Supplementary material for: Mapping the evolution of fertility support policies in China: A content and instrumental analysis
Source: PLoS One. 2025 Oct 9;20(10):e0332137. doi: 10.1371/journal.pone.0332137 (PMC12510515; doi:10.1371/journal.pone.0332137)
Supplement: S1 Appendix — (ZIP) [file pone.0332137.s001.zip › S1 Appendix. 226 original policy documents/44-国家卫生健康委员会、国家发展和改革委员会、中共中央宣传部等关于进一步完善和落实积极生育支持措施的指导意见(FBM-CLI-4-5132874).doc]

国家卫生健康委员会、国家发展和改革委员会、中共中央宣传部等关于进一步完善和落实积极生育支持措施的指导意见

**发布部门：** [国家卫生健康委员会](https://www.pkulaw.com/law/chl?Aggs.IssueDepartment=60321) [国家发展和改革委员会(含原国家发展计划委员会、原国家计划委员会)](https://www.pkulaw.com/law/chl?Aggs.IssueDepartment=60301) [中共中央宣传部](https://www.pkulaw.com/law/chl?Aggs.IssueDepartment=a04) [教育部](https://www.pkulaw.com/law/chl?Aggs.IssueDepartment=60203) [民政部](https://www.pkulaw.com/law/chl?Aggs.IssueDepartment=60208) [财政部](https://www.pkulaw.com/law/chl?Aggs.IssueDepartment=60210) [人力资源和社会保障部](https://www.pkulaw.com/law/chl?Aggs.IssueDepartment=60236) [住房和城乡建设部](https://www.pkulaw.com/law/chl?Aggs.IssueDepartment=60238) [中国人民银行](https://www.pkulaw.com/law/chl?Aggs.IssueDepartment=60701) [国务院国有资产监督管理委员会](https://www.pkulaw.com/law/chl?Aggs.IssueDepartment=60305) [国家税务总局](https://www.pkulaw.com/law/chl?Aggs.IssueDepartment=60401) [国家医疗保障局](https://www.pkulaw.com/law/chl?Aggs.IssueDepartment=60431) [中国银行保险监督管理委员会](https://www.pkulaw.com/law/chl?Aggs.IssueDepartment=60322) [中华全国总工会](https://www.pkulaw.com/law/chl?Aggs.IssueDepartment=70401) [共青团中央](https://www.pkulaw.com/law/chl?Aggs.IssueDepartment=70402) [全国妇女联合会](https://www.pkulaw.com/law/chl?Aggs.IssueDepartment=70403) [中央军委后勤保障部](https://www.pkulaw.com/law/chl?Aggs.IssueDepartment=b15) [机构沿革](javascript:void(0))

**发文字号：**国卫人口发〔2022〕26号

**发布日期：**2022.07.25

**实施日期：**2022.07.25

**时效性：** [现行有效](https://www.pkulaw.com/law/chl?Aggs.TimelinessDic=01)

**效力级别：** [部门规范性文件](https://www.pkulaw.com/law/chl?Aggs.EffectivenessDic=XE0302)

**法规类别：** [计划生育管理](https://www.pkulaw.com/law/chl?Aggs.Category=09705)

国家卫生健康委员会、国家发展和改革委员会、中共中央宣传部、教育部、民政部、财政部、人力资源和社会保障部、住房和城乡建设部、中国人民银行、国务院国有资产监督管理委员会、国家税务总局、国家医疗保障局、中国银行保险监督管理委员会、中华全国总工会、中国共产主义青年团中央委员会、中华全国妇女联合会、中央军委后勤保障部关于进一步完善和落实积极生育支持措施的指导意见
（国卫人口发〔2022〕26号）

各省、自治区、直辖市人民政府，国务院各部委、各直属机构，军队各有关单位：
　　为深入贯彻《中共中央 国务院关于[优化生育政策促进人口长期均衡发展的决定](https://www.pkulaw.com/chl/1247bc81dccaeed3bdfb.html)》，落实[人口与计划生育法](https://www.pkulaw.com/chl/d560ce000fba464cbdfb.html)，进一步完善和落实积极生育支持措施，经国务院同意，现提出如下意见：
　　**一、**总体要求
　　坚持以习近平新时代中国特色社会主义思想为指导，认真贯彻落实党中央、国务院决策部署，深入实施一对夫妻可以生育三个子女政策及配套支持措施，将婚嫁、生育、养育、教育一体考虑，尽力而为、量力而行，综合施策、精准发力，完善和落实财政、税收、保险、教育、住房、就业等积极生育支持措施，落实政府、用人单位、个人等多方责任，持续优化服务供给，不断提升服务水平，积极营造婚育友好社会氛围，加快建立积极生育支持政策体系，健全服务管理制度，为推动实现适度生育水平、促进人口长期均衡发展提供有力支撑。

　　**二、**提高优生优育服务水平
　　（一）改善优生优育全程服务。实施母婴安全行动提升计划，全面落实母婴安全五项制度。推进妇幼保健机构能力建设，各省、市、县级均应设置1所政府举办、标准化的妇幼保健机构。加强高质量产科建设，全面改善住院分娩条件。推动落实出生缺陷三级防治策略，健全“县级筛查、市级诊断、省级指导、区域辐射”的出生缺陷防治网络，提升婚前保健、孕前保健、产前筛查和产前诊断服务水平，针对重点疾病推动围孕期、产前产后一体化管理服务和多学科诊疗协作，强化新生儿遗传代谢病、听力障碍和先天性心脏病筛查和诊断。
　　（二）提高儿童健康服务质量。实施健康儿童行动提升计划。加强0-6岁儿童和孕产妇健康管理服务，提高服务质量和资金使用效率。加强基层儿童保健服务网络建设。推进基层医疗机构儿童保健门诊（儿童保健室）标准化建设，提高乡镇卫生院、社区卫生服务中心专业从事儿童保健和基本医疗服务的医生配备水平。“十四五”期间，中央预算内投资支持开展10个左右儿科类国家区域医疗中心建设项目，推进儿科医疗联合体建设，促进优质儿科医疗资源下沉和均衡布局。开展母婴友好医院和儿童友好医院建设。做好新生儿参加居民医保服务管理工作。
　　（三）加强生殖健康服务。扩大分娩镇痛试点，规范相关诊疗行为，提升分娩镇痛水平。指导推动医疗机构通过健康教育、心理辅导、中医药服务、药物治疗、手术治疗、辅助生殖技术等手段，向群众提供有针对性的服务，提高不孕不育防治水平。推进辅助生殖技术制度建设，健全质量控制网络，加强服务监测与信息化管理。开展生殖健康促进行动，增强群众保健意识和能力。加强生殖健康宣传教育和服务，预防非意愿妊娠，减少非医学需要的人工流产。
　　（四）提高家庭婴幼儿照护能力。建立完善健康科普专家库和资源库，通过广播、电视、报刊、网络、新媒体等多种渠道，普及科学育儿知识与技能。鼓励地方采取积极措施，支持隔代照料、家庭互助等照护模式。扩大家政企业上门居家婴幼儿照护服务供给。鼓励有条件的托育机构与家政企业等合作，提供上门居家婴幼儿照护服务。鼓励有资质的服务机构、行业协会和专业人员，依托村（居）委会等基层力量，通过家长课堂、养育照护小组活动、入户指导等方式，提高婴幼儿照护能力。充分发挥公益慈善类社会组织等社会力量积极作用，加大对农村和欠发达地区婴幼儿照护服务的支持。

　　**三、**发展普惠托育服务体系
　　（五）增加普惠托育服务供给。2022年，全国所有地市要印发实施“一老一小”整体解决方案。通过中央预算内投资支持和引导，实施公办托育服务能力建设项目和普惠托育服务专项行动，带动地方政府基建投资和社会投资。公办托育机构收费标准由地方政府制定，加强对普惠托育机构收费的监管。拓展社区托育服务功能，完善婴幼儿照护设施等基本公共服务设施。支持有条件的用人单位为职工提供福利性托育服务。加快制定出台家庭托育点管理办法。在满足学前教育普及的基础上，鼓励和支持有条件的幼儿园招收2-3岁幼儿。
　　（六）降低托育机构运营成本。“十四五”时期，拓宽托育建设项目申报范围，中央预算内投资加大支持力度给予建设补贴。科学布局社区综合服务设施，落实社区托育服务发展税费优惠政策。完善土地、住房、财政、金融、人才等政策，鼓励地方对普惠托育机构予以支持。托育机构用水用电用气用热按照居民生活类价格执行。鼓励社会资本设立托育服务事业发展基金，向托育行业提供增信支持。各地要建立托育机构关停等特殊情况应急处置机制，落实疫情期间托育企业纾困政策。
　　（七）提升托育服务质量。深入开展全国婴幼儿照护服务示范城市创建活动，形成一批可复制、可推广的典型经验。研究制定托育服务相关制度规范，大力发展多种形式的托育服务。鼓励有条件的普通高等学校和职业院校开设托育服务相关专业，加快培养专业人才。依法逐步实行托育从业人员职业资格准入制度。深入实施康养职业技能培训计划，加强托育岗位人员技能培训。各级医疗卫生机构、疾病预防控制机构等要加强对托育机构卫生保健工作的业务指导、咨询服务和监督检查，预防控制传染病，降低常见病的发病率，保障婴幼儿的身心健康。严格落实托育机构消防安全指南等一系列规范性文件，加强部门综合监管，严防安全事故发生。加强社会监督，促进行业自律。

　　**四、**完善生育休假和待遇保障机制
　　（八）优化生育休假制度。各地要完善生育休假政策，从保障职工生育权益和保护生育职工健康权的功能定位出发，体现保护生育和养育过程，帮助职工平衡工作和家庭关系，促进公平就业和职业发展。要结合实际完善假期用工成本合理分担机制，明确相关各方责任，采取切实有效措施保障职工假期待遇。
　　（九）完善生育保险等相关社会保险制度。国家统一规范并制定完善生育保险生育津贴支付政策，强化生育保险对参保女职工生育医疗费用、生育津贴待遇等保障作用，保障生育保险基金安全。有条件的地方可探索参加职工基本医疗保险的灵活就业人员同步参加生育保险。未就业妇女通过参加城乡居民基本医疗保险享受生育医疗待遇。为领取失业保险金人员缴纳职工基本医疗保险费（含生育保险费），保障其生育权益，所需资金从失业保险基金列支。指导地方综合考虑医保（含生育保险）基金可承受能力、相关技术规范性等因素，逐步将适宜的分娩镇痛和辅助生殖技术项目按程序纳入基金支付范围。

　　**五、**强化住房、税收等支持措施
　　（十）加快完善住房保障体系。加快发展保障性租赁住房，促进解决新市民、青年人等群体住房困难。进一步完善公租房保障对促进积极生育的支持措施，各地在配租公租房时，对符合条件且有未成年子女的家庭，可根据其未成年子女数量，在户型选择方面给予适当照顾；优化公租房轮候与配租规则，将家庭人数及构成等纳入轮候排序或综合评分的因素，对符合条件且子女数量较多的家庭可直接组织选房；完善公租房调换政策，对因家庭人口增加、就业、子女就学等原因需要调换公租房的，根据房源情况及时调换。
　　（十一）精准实施购房租房倾斜政策。住房政策向多子女家庭倾斜，在缴存城市无自有住房且租赁住房的多子女家庭，可按照实际房租支出提取住房公积金；对购买首套自住住房的多子女家庭，有条件的城市可给予适当提高住房公积金贷款额度等相关支持政策。加快发展长租房市场，多渠道增加长租房供应，推进租购权利均等。各地可结合实际，进一步研究制定根据养育未成年子女负担情况实施差异化租赁和购买房屋的优惠政策。
　　（十二）发挥好税收、金融等支持作用。实施好3岁以下婴幼儿照护费用个人所得税专项附加扣除政策。建立对依法保障职工生育权益用人单位激励机制。向提供母婴护理、托育服务以及相关职业培训、消费品生产的企业加大金融支持力度。

　　**六、**加强优质教育资源供给
　　（十三）提高学前教育普及普惠水平。继续实施“十四五”学前教育发展提升行动计划，着力补齐农村地区和城市新增人口集中地区普惠性资源短板。切实落实各级政府发展学前教育责任，健全政府投入为主、家庭合理分担、其他多渠道筹措经费的机制。优化完善财政补助政策，逐步提高学前教育财政投入水平，保障普惠性学前教育有质量可持续发展。健全学前教育资助制度，切实保障家庭经济困难儿童接受普惠性学前教育。
　　（十四）提高义务教育均衡发展水平。依法落实政府举办义务教育的主体责任，优化义务教育结构，确保义务教育学位主要由公办学校提供和政府购买学位方式提供。继续落实“两免一补”政策，降低学生就学成本。进一步减轻义务教育阶段学生作业负担和校外培训负担，发挥学校教育主阵地作用，提升课后服务质量，按规定保障课后服务经费。严格落实义务教育阶段学科类校外培训收费实行政府指导价管理政策。加强非学科类校外培训监管，规范培训机构收费行为。加强对家长的家庭教育指导，树立科学育儿观念。
　　（十五）加强生理卫生等健康教育。针对在校学生的心理生理特点，通过定期举办专题讲座、开设公共选修课程等方式，开展生理卫生教育、青春期教育或者性健康教育，加强婚恋观、家庭观正向引导。

　　**七、**构建生育友好的就业环境
　　（十六）鼓励实行灵活的工作方式。用人单位可结合生产和工作实际，通过与职工协商，采取弹性上下班、居家办公等工作方式，为有接送子女上下学、照顾生病或居家子女等需求的职工提供工作便利，帮助职工解决育儿困难。
　　（十七）推动创建家庭友好型工作场所。推动用人单位将帮助职工平衡工作和家庭关系相关措施纳入集体合同和女职工权益保护专项集体合同条款。实施母乳喂养促进行动。女职工比较多的用人单位应当建立孕妇休息室、哺乳室，配备必要母婴服务设施，更好满足孕产期、哺乳期女职工的需求。鼓励有条件的用人单位、学校、社区、群团组织等开展寒暑假托管服务。
　　（十八）切实维护劳动就业合法权益。推动完善促进妇女就业的制度机制，加强对女性劳动者特别是生育再就业女性相关职业技能培训。持续开展就业性别歧视约谈工作，依法查处侵权行为。督促用人单位依法依规落实对孕产期、哺乳期女职工关于工作时间、工资待遇、劳动强度等方面的特殊劳动保护。加强监管执法，健全司法救济机制，探索开展妇女平等就业权益保护检察公益诉讼，维护妇女劳动和社会保障权益。强化工会劳动法律监督，推动职工权益保护法律法规贯彻落实。

　　**八、**加强宣传引导和服务管理
　　（十九）积极营造生育友好社会氛围。充分发挥各类媒体作用和群团组织优势，积极开展人口基本国情宣传教育，弘扬中华民族传统美德，提倡适龄婚育、优生优育，倡导尊重生育的社会价值、尊重父母、儿童优先、夫妻共担育儿责任。推进婚俗改革和移风易俗，破除婚嫁大操大办、高价彩礼等陈规陋习，倡导积极婚育观念。组织创作一批积极向上的文艺作品，讲好新时代美好爱情、和谐家庭、幸福生活的中国故事。推进儿童友好城市建设。开展全国生育友好工作先进单位表彰活动，评选一批工作扎实、成效明显、群众满意的先进典型，鼓励和带动基层积极创新，营造生育友好的社会环境。
　　（二十）建立健全人口服务体系。以“一老一小”为重点，建立健全覆盖全生命周期的人口服务体系。加强政府和社会协同治理，强化乡镇（街道）、村（社区）等基层人口管理体系和服务能力建设。完善生育登记制度，全面落实出生医学证明、儿童预防接种、户口登记、医保参保、社保卡申领等“出生一件事”集成化办理。强化基层人口信息管理职责，促进入户、入学、婚姻登记、卫生健康等基础信息融合共享，科学研判生育形势和人口变动趋势。维护计划生育家庭合法权益，建立健全政府主导、社会组织参与的计划生育特殊家庭扶助关怀工作机制，建立健全生活、养老、医疗、精神慰藉等全方位帮扶保障制度，动员各级计划生育协会深入开展“暖心行动”。
　　各地各有关部门要深刻认识完善和落实积极生育支持措施的重要性和紧迫性，切实提高政治站位，把人口工作摆上重要议事日程，密切协同配合，加快完善积极生育支持措施。各地要坚持一把手亲自抓、负总责，加强统筹规划、政策协调和工作落实，结合实际及时完善具体政策措施，周密组织实施，确保责任到位、措施到位、投入到位、落实到位。各有关部门要加强对地方的指导，总结推广好的经验做法，及时细化配套措施，推动解决工作中面临的问题，不断完善服务管理制度。立足国情，加强评估论证，促进生育政策和相关经济社会政策配套衔接。完善跨部门协调机制，强化重要政策统筹研究和督促落实。完善优化生育政策目标管理责任制，研究建立指标体系，监测评估积极生育支持措施的成效。加强政策宣传解读，及时妥善回应社会关切，营造良好氛围。重要情况及时报告。

国家卫生健康委
国家发展改革委
中央宣传部
教育部
民政部
财政部
人力资源社会保障部
住房城乡建设部
中国人民银行
国务院国资委
国家税务总局
国家医保局
中国银保监会
全国总工会
共青团中央
全国妇联
中央军委后勤保障部
2022年7月25日

© 北大法宝：（[www.pkulaw.com](https://www.pkulaw.com/)) 专业提供法律信息、法学知识和法律软件领域各类解决方案。北大法宝为您提供丰富的参考资料，正式引用法规条文时请与标准文本核对。
欢迎查看所有[产品和服务](https://www.pkulaw.com/helps/2.html)。法宝快讯：[如何快速找到您需要的检索结果？](https://www.pkulaw.com/helps/70.html)[法宝](https://www.pkulaw.com/helps/69.html)V6有何新特色？

扫描二维码阅读原文

**原文链接：**<https://www.pkulaw.com/chl/cce9b2093738a372bdfb.html>
